# Metabolic flux analysis of coenzyme Q_10_ synthesized by *Rhodobacter sphaeroides* under the influence of different pH regulators

**DOI:** 10.1186/s12934-023-02205-z

**Published:** 2023-10-10

**Authors:** Yujun Xiao, Yi Zheng, Yong Zhou, Chaofan Yu, Ting-E Ye

**Affiliations:** National Joint Engineering Research Center of Industrial Microbiology and Fermentation Technology, College of Life Sciences, Fujian Normal University, Fuzhou, China

**Keywords:** CoQ_10_, Metabolic flux, Metabolic engineering, *Rhodobacter sphaeroides*

## Abstract

Coenzyme Q_10_ (CoQ_10_) is crucial for human beings, especially in the fields of biology and medicine. The aim of this experiment was to investigate the conditions for increasing CoQ_10_ production. At present, microbial fermentation is the main production method of CoQ_10_, and the production process of microbial CoQ_10_ metabolism control fermentation is very critical. Metabolic flux is one of the most important determinants of cell physiology in metabolic engineering. Metabolic flux analysis (MFA) is used to estimate the intracellular flux in metabolic networks. In this experiment, *Rhodobacter sphaeroides* was used as the research object to analyze the effects of aqueous ammonia (NH_3_·H_2_O) and calcium carbonate (CaCO_3_) on the metabolic flux of CoQ_10_. When CaCO_3_ was used to adjust the pH, the yield of CoQ_10_ was 274.43 mg·L^−1^ (8.71 mg·g^−1^ DCW), which was higher than that of NH_3_·H_2_O adjustment. The results indicated that when CaCO_3_ was used to adjust pH, more glucose-6-phosphate (G6P) entered the pentose phosphate (HMP) pathway and produced more NADPH, which enhanced the synthesis of CoQ_10_. At the chorismic acid node, more metabolic fluxes were involved in the synthesis of p-hydroxybenzoic acid (pHBA; the synthetic precursor of CoQ_10_), enhancing the anabolic flow of CoQ_10_. In addition, Ca^2+^ produced by the reaction of CaCO_3_ with organic acids promotes the synthesis of CoQ_10_. In summary, the use of CaCO_3_ adjustment is more favorable for the synthesis of CoQ_10_ by *R. sphaeroides* than NH_3_·H_2_O adjustment. The migration of metabolic flux caused by the perturbation of culture conditions was analyzed to compare the changes in the distribution of intracellular metabolic fluxes for the synthesis of CoQ_10_. Thus, the main nodes of the metabolic network were identified as G6P and chorismic acid. This provides a theoretical basis for the modification of genes related to the CoQ_10_ synthesis pathway.

## Introduction

**Fig. 1:**
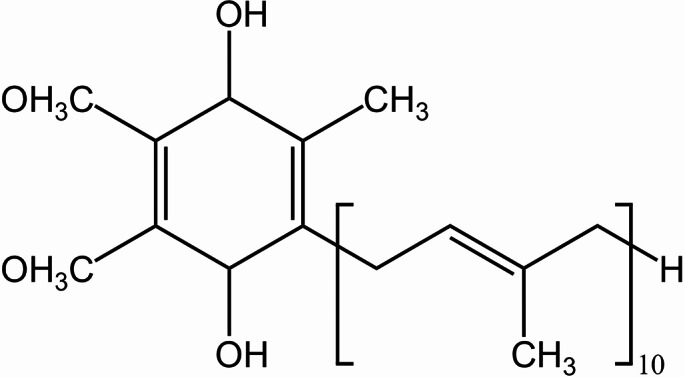
Chemical structure of reduced CoQ_10_ (CoQ_10_H_2_)

Coenzyme Q_n_ (CoQ_n_) is a lipid-soluble quinone compound formed by conjugation of the benzoquinone group at the head and a polyisoprene hydrophobic side chain at the tail [1]. It exists in the cells of various organisms. The subscript n of CoQ_n_ represents the number of isoprene repeating units in the molecule. The number of isoprene units on the side chains of CoQ_n_ varies from organism to organism. The CoQ_n_ molecule in the human body contains 10 isoprene units, namely CoQ_10_. CoQ_10_ was discovered by Frederick Crane and colleagues in 1957 [2]. CoQ_10_ is the only lipid-soluble antioxidant synthesized endogenously in the human body [3]. Reduced CoQ_10_ (Fig. 1) is the main form present in the body, accounting for more than 90% of the total CoQ_10_ in the human circulation [4, 5]. The reduced CoQ_10_ releases electrons during the electron transfer process, and the electrons are transferred through the respiratory chain to eventually produce ATP to provide energy for the body. CoQ_10_ deficiency in the human body will cause some diseases. In addition, CoQ_10_ is also beneficial to the skin. It can improve the skin and enhance the expression of collagen and elastin [6, 7]. It has been shown that CoQ_10_ levels in skin and skin surface lipids decline with age. CoQ_10_ is very important to human beings and has great value. It is used in many fields such as medicine, food, and cosmetics.

There are three main methods to produce CoQ_10_. However, there is a preference for the microbial fermentation method, which is considered the most feasible, over the biological tissue extraction method and chemical synthesis method [8–10]. CoQ_10_ synthesized by microbial fermentation is an all-trans conformation with biological activity, and the method does not produce other optical isomers [11, 12]. In addition, microorganisms grow and reproduce quickly, can utilize inexpensive raw materials, and produce fewer contaminants during the production process. One of the microorganisms that can synthesize CoQ_10_ efficiently and has been used in industrial production is *Rhodobacter sphaeroides* [13, 14]. It is a gram-negative purple non sulfur bacterium (PNSB) that can grow aerobically, anaerobically, photoautotrophically, and heterotrophically [15–17]. It has many other functions, such as biological hydrogen production, synthesis of carotenoids, fixation of CO_2_ and N_2_, remediation of heavy metal pollution and power generation [18–21].

Extracellular organic acids are continuously produced by *R. sphaeroides* during the fermentation of CoQ_10_. This leads to a continuous decrease in the pH of the environment and affects the physiological activity of the bacteria. Organic acids are generally neutralized by adding CaCO_3_ or NH_3_·H_2_O.

**Fig. 2:**
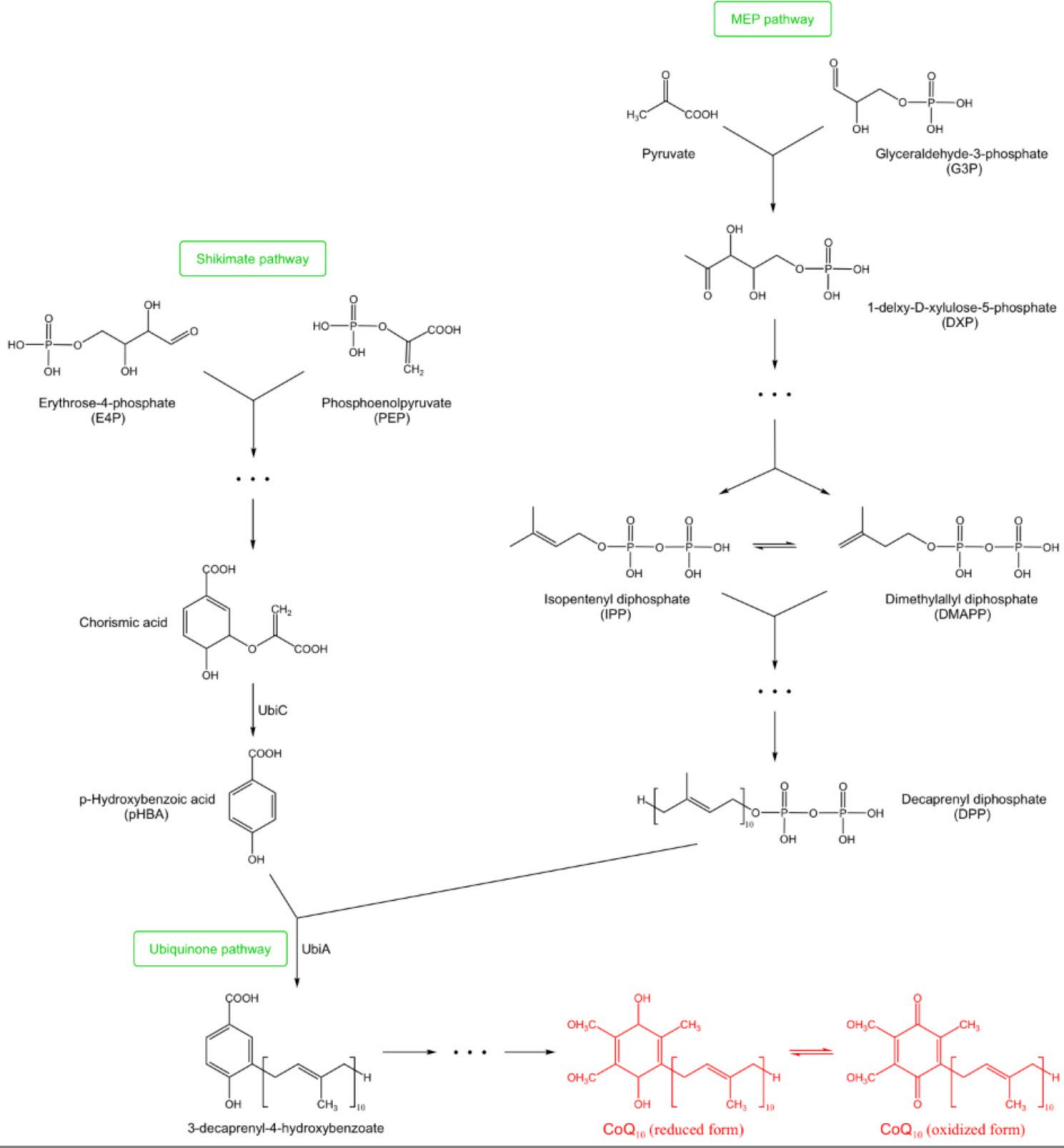
CoQ_10_ synthesis pathway in *R. sphaeroides*

The process of CoQ_10_ biosynthesis in *R. sphaeroides* is complex (Fig. 2). First, the head quinone ring and the tail polyisoprene chain of CoQ_10_ need to be synthesized separately [22]. The synthesis of the quinone ring is the shikimate pathway. This pathway uses erythrose-4-phosphate (E4P) and phosphoenolpyruvate (PEP) as the starting substrates, which are converted to chorismic acid in various steps. The chorismic acid is then converted to p-hydroxybenzoic acid (pHBA), a precursor of the quinone ring, by the action of the chorismic acid lyase encoded by the UbiC gene. The polyisoprene chain is synthesized via the methylerythritol phosphate (MEP) pathway. The starting substances of this pathway are glyceraldehyde-3-phosphate and pyruvate, which are converted to isopentenyl diphosphate (IPP) and dimethylallyl diphosphate (DMAPP) by various reactions, and then formed into decaprenyl diphosphate (DPP) by various reactions. DPP and pHBA are converted to 3-decaprenyl-4-hydroxybenzoate via the ubiquinone pathway by the action of p-hydroxybenzoic acid polyisoprene transferase encoded by the UbiA gene and enter the quinone ring modification pathway. After decarboxylation, hydroxylation, and methylation processes, CoQ_10_ is finally formed.

## Methods and materials

### Microorganism and culture media

The *R. sphaeroides* used in this study was the F3-40 strain (from the College of Life Sciences, Fujian Normal University, Fuzhou, China). The culture media included agar slant culture medium (containing 18 g·L^− 1^ glucose, 4.5 g·L^− 1^ yeast powder, 20 g·L^− 1^ agar powder, 2 g·L^− 1^ monosodium glutamate, 5.25 g·L^− 1^ (NH_4_)_2_SO_4_, 4.2 g·L^− 1^ NaCl, 8.75 g·L^− 1^ MgSO_4_, 1.5 g·L^− 1^ KH_2_PO_4_, 1.2 g·L^− 1^ A), seed culture medium (containing 18 g·L^− 1^ glucose, 4.5 g·L^− 1^ yeast powder, 2 g·L^− 1^ monosodium glutamate, 5.25 g·L^− 1^ (NH_4_)_2_SO_4_, 4.2 g·L^− 1^ NaCl, 8.75 g·L^− 1^ MgSO_4_, 1.5 g·L^− 1^ KH_2_PO_4_, 1.2 g·L^− 1^ A), and fermentation culture medium (containing 35.6 g·L^− 1^ glucose, 6.39 g·L^− 1^ corn steep powder, 7.39 g·L^− 1^ monosodium glutamate, 6.28 g·L^− 1^ (NH_4_)_2_SO_4_, 2.5 g·L^− 1^ NaCl, 12 g·L^− 1^ MgSO_4_, 0.8 g·L^− 1^ KH_2_PO_4_, 0.1 g·L^− 1^ MnSO_4_, 1.6 g·L^− 1^ A, 0.1 g·L^− 1^ B).

### Cultivation method

Two colonies of *R. sphaeroides* cultured on agar plates for 6 days were picked out and transferred into the primary seed medium (50 mL/250 mL), and incubated at 32 °C with shaking at 220 rpm for 24 h. Then, they were transferred into the secondary seed medium (350 mL/2500 mL) at 10% inoculum and incubated at 32 °C with shaking at 220 rpm for 22 h. Finally, the secondary seed medium was inoculated at 15% inoculum into a 10 L fermenter. The inoculated fermenter was filled with 7 L. The temperature was 34 °C, the stirring speed was 300 rpm, the aeration rate was 3 L·min^− 1^, and the tank pressure was 0.04 MPa. The pH was adjusted by adding CaCO_3_ and NH_3_·H_2_O during the fermentation process. Samples were taken at 3 h intervals to determine various parameters.

### Determination of glucose

A bioanalytical sensor (model SBA-40D, from Biology Institute of Shandong Academy of Sciences) was used for analysis. The fermentation broth was diluted by a certain multiple and then filtered. Twenty-five microliters of filtrate was taken for injection determination. The determination was performed three times in parallel and the average value was taken.

### Determination of cell dry weight (DCW)

Five milliliters of the fermentation broth was centrifuged (1 × 10^4^ rpm, 10 min), and the supernatant was removed. After washing with distilled water, the samples were centrifuged again, and the supernatant was removed. The organism in the centrifuge tube was dried in an oven at 100 °C to a constant weight. For fermentation broth containing CaCO_3_, a drop of 6 mol·L^− 1^ HCl solution was added to dissolve the residual CaCO_3_ in the fermentation broth before centrifugation.

### Extraction of CoQ_10_ and determination of its content

CoQ_10_ was extracted by ultrasonic extraction [23, 24]. Five milliters of the fermentation broth was added to a 50 mL brown volumetric flask. A drop of 6 mol·L^− 1^ HCl solution was added. Shake well and add 10 mL of acetone. After mixing, 0.5 mL of 30% H_2_O_2_ solution was added. Finally, 20–30 mL ethanol was added. The volumetric flask was placed into the ultrasonic cleaner for 1 min, and the volumetric flask was not capped. Subsequently, the volumetric flask was fixed with ethanol and capped. A strip of cardboard was used to separate the cap from the neck of the bottle to retain the gap. The volumetric flask was again placed in an ultrasonic cleaner for 45 min, with the temperature controlled below 35 °C. At the end of the treatment, the flask was well shaken and allowed to stand for 30 min. Finally, the supernatant was filtered through a 0.22 μm organic filter.

The filtrate was collected for the determination of CoQ_10_ by high-performance liquid chromatography (HPLC). The chromatographic column was Hypersil ODS-SP (4.6 mm × 100 mm, 5 μm). The mobile phase consisted of anhydrous methanol and anhydrous ethanol in a ratio of 65: 35 (v/v). The detection wavelength was set at 275 nm. The column temperature was 30 °C, the injection volume was 20 µL, the flow rate was 1.1 mL·min^− 1^, and the elution time was 15 min. The calculation formula of CoQ_10_ concentration in the sample is:

$$c = \frac{{S \cdot {V_o} \cdot K \cdot {c_o}}}{{{S_o} \cdot V}}$$

where c is the concentration of CoQ_10_ in the test sample (mg·L^− 1^); c_o_ is the concentration of CoQ_10_ in the standard sample (mg·L^− 1^); K is the dilution multiple of the test sample; S is the peak area of the test sample (mAU·s); S_o_ is the peak area of the standard sample (mAU·s); V is the injection volume of the test sample (µL); and V_o_ is the injection volume of the standard sample (µL).

### Determination of various free amino acid concentrations

The concentration of free amino acids in the fermentation broth was determined by HPLC. A Venusil-AA amino acid analysis column was used. Mobile phases A and B were 0.1 mol·L^− 1^ CH_3_COONa solution (pH 6.5) and 80% acetonitrile solution, respectively. The detection wavelength was set at 254 nm. The column temperature was 40 °C, the injection volume was 20 µL, and the flow rate was 1 ml·min^− 1^. Gradient elution was performed.

### Determination of organic acid concentration

The concentration of organic acids in the fermentation broth was determined by HPLC [25]. The chromatographic column was an Inertsil ODS-SP C18. Mobile phases A and B were 0.01 mol·L^− 1^ KH_2_PO_4_ buffer (pH adjusted to 2.8 by H_3_PO_4_) and acetonitrile solution, respectively, and were injected into the column at a ratio of 95: 5 (v/v). The detection wavelength was set at 215 nm. The column temperature was 25 °C, the injection volume was 20 µL, and the flow rate was 1 ml·min^− 1^.

## Results

### Construction of a metabolic network for CoQ_10_ synthesis in *R. sphaeroides*

The synthesis of CoQ_10_ by *R. sphaeroides* belongs to growth-coupled fermentation. Therefore, when analyzing the metabolic flux of CoQ_10_ synthesis, the carbon source consumed by the growth of the bacterium cannot be ignored, and the synthesis pathway of the basic cellular components should be considered. The main cellular components and their contents refer to the experimental data measured by Saheed lmam [26]. The fractions of *R. sphaeroides* under aerobic conditions are shown in Table 1. It contained 17.6% pHBA. Among fatty acids, oleic acid (C_18_:1) has the highest content, accounting for 85%. Stearic acid (C_18_:0), soft fatty acid (C_16_:0), and palmitoleic acid (C_16_:1) accounted for 9%, 5%, and 1%, respectively.

**Table 1:** Distribution of cellular fraction components of *R. sphaeroides* under aerobic contributions

Component	Content (mmol·g^− 1^ DCW)	Component	Content (mmol·g^− 1^ DCW)	Component	Content (mmol·g^− 1^ DCW)
Ala	0.8189	Lys	0.1669	CTP	0.0904
Arg	0.431	Met	0.1585	UTP	0.0436
Asn	0.1423	Phe	0.2041	dATP	0.0185
Asp	0.3344	Pro	0.3242	dTTP	0.0186
Cys	0.0714	Ser	0.2738	dGTP	0.0412
Gln	0.1603	Thr	0.3134	dCTP	0.0415
Glu	0.401	Trp	0.063	PE	0.077
Gly	0.5535	Tyr	0.1207	PG	0.0867
His	0.1363	Val	0.4527	PC	0.0385
Ile	0.2906	ATP	0.0455	DPG	0.0024
Leu	0.5511	GTP	0.0932	SQDG	0.0241

Since *R. sphaeroides* uses glucose as the sole carbon source, the glyoxylate cycle pathway is largely inoperative and is not considered in this metabolic network. The intermediate reactions without branching points are simplified into a reaction equation. For intermediate metabolites with branching points, the rate of production was equal to the rate of consumption, and the net accumulation was 0, assuming that these metabolites were in the proposed steady state.

It is assumed that NADPH produced by the HMP pathway and the TCA pathway were not oxidatively phosphorylated but were all used for biosynthesis. The fermentation supernatant of 39–42 h in the late logarithmic growth phase of the organism was analyzed for composition. The results showed that the supernatant basically did not contain free amino acids. The content of organic acid metabolic byproducts such as formic acid, acetic acid, propionic acid, butyric acid, lactic acid, pyruvic acid, and citric acid was also very low. Therefore, the influence of these organic acids on metabolic flux can be ignored in the metabolic network studied.

### Modeling of metabolic flux equilibrium for CoQ_10_ synthesis by *R. sphaeroides*

Metabolic flux analysis is a metabolic network chemometric used to describe the conversion of substrates into metabolites and cellular composition. The distribution of carbon flow under the condition of carbon source limitation is generally considered. The equilibrium equation of each metabolite can be expressed as:

$$\frac{d{X}_{met}}{dt}={r}_{met}-u{X}_{met}$$

where X_met_ is the metabolite concentration; r_met_ is the net rate of metabolite formation; and u is the dilution factor of the culture medium.

Since most of the intermediate metabolites have low intracellular concentrations, the dilution effect can be negligible. According to the proposed steady-state assumption that the concentration of intermediate metabolites is in equilibrium (dX_met_/dt = 0), it follows that: r_met_ = 0.

The net rate of intermediate metabolite formation used is expressed in the form of a matrix equation as:

$$S \cdot v = 0$$

where S is the n × m stoichiometric coefficient matrix, and the rows of the matrix represent the stoichiometric coefficients of the rate reactions of the intermediate metabolites; v is an m-dimensional column vector; n is the number of intermediate metabolites; and m is the total number of metabolic reaction rates.

The degree of freedom to be solved is F = m - n. The number of linearly uncorrelated rates is determined experimentally. The matrix equation S · v = 0 has a unique solution as long as it is greater than or equal to the degree of freedom F. Then, the undetermined metabolic reaction rates can be calculated, and thus, the flux distribution of the metabolic network can be determined.

Based on the intracellular composition and biosynthetic analysis of the various metabolites in the metabolic network, the following proposed steady-state equilibrium equation of the branch point intermediates was obtained:

G6P: r_1_-r_2_-r_10_-r_11_-0.0241r_21_ = 0.

F6P: r_2_-r_3_ + 0.6667r_13_ + r_14_ = 0.

Ru5P: r_11_-0.5288r_12_-r_13_-2r_14_-0.063r_15_ = 0.

GA3P: 2r_3_-r_4_ + 0.3333r_13_ + 0.063r_15_-10r_16_-0.313r_21_ = 0.

PG: r_4_-r_5_-0.1984r_12_-0.8987r_20_-0.1155r_21_ = 0.

PEP: r_5_-r_6_-2r_14_-0.063r_15_ = 0.

Pyr: r_6_-r_7_ + 0.063r_15_-9r_16_-2.0895r_17_-r_18_-0.4575r_19_ = 0.

AcCoA: r_7_-r_8_-0.5511r_17_-8.0862r_21_ = 0.

OAA: -r_8_ + r_9_-0.1941r_12_ + r_18_-1.4061r_19_ = 0.

Chr: r_14_-0.3878r_15_-r_16_ = 0.

AKG: r_8_-r_9_-1.3165r_22_ = 0.

NADPH: r_8_ + 2r_11_-0.911r_12_-r_14_-0.3878r_15_-10r_16_-2.0895r_17_-4.8055r_19_-1.1843r_20_-7.3028r_21_-3.2579r_22_ = 0.

When these equilibrium equations were constructed into a matrix equation, the rank of the matrix n = 12 was calculated. If the unknown rate variable J = 22, then the matrix has degrees of freedom F = J - n = 10. If the matrix equation is to have a unique solution, at least 10 rate variables must be determined. Of these 22 rate variables, r_10_, r_12_, r_15_, r_17_, r_19_, r_20_, r_21_, and r_22_ were calculated by measuring the synthesis rate of cell dry weight. Through the sugar consumption rate, r_1_ can be obtained. Through the synthesis rate of CoQ_10_, r_16_ can be obtained. From the data of these 10 rate variables, the remaining 12 unknown rate variables can be calculated using MATLAB software.

### Analysis of the effects of two pH regulators on the synthetic metabolic flux of CoQ_10_

The synthesis rate of CoQ_10_ was significantly higher in the late logarithmic growth phase than in the mid and early growth periods when the pH was adjusted by CaCO_3_ or NH_3_·H_2_O. Then, metabolic flux analysis was performed to quantify the flux distribution of the CoQ_10_ synthesis pathway in the late logarithmic growth phase of these two fermentations. Flux analysis was performed using the 42nd hour of fermentation as the time point. The variable rates of metabolites (r_1_ to r_22_) were divided by the rate of glucose consumption (r_1_), and the result was the respective values of r1 to r22 (Table 2).

**Table 2:** The metabolic flux distribution of CoQ_10_ under different pH adjustment modes

Flux	CaCO_3_ adjustment	NH_3_·H_2_O adjustment	C (%)
r1	100	100	0
r2	27.88	30.45	-8.44
r3	73.56	74.46	-1.21
r4	82.62	83.42	-0.96
r5	79.56	80.38	-1.02
r6	78.63	79.51	-1.11
r7	60.73	62	-2.05
r8	8.06	9.15	-11.91
r9	12.6	15.37	-18.02
r10	1.29	1.28	0.78
r11	70.54	67.99	3.75
r12	1.22	1.21	0.83
r13	56.02	54.01	3.72
r14	1.44	1.33	8.27
r15	1.31	1.3	0.77
r16	0.13	0.03	333.33
r17	5.94	5.9	0.68
r18	9.99	9.91	0.81
r19	6.9	6.85	0.73
r20	2.12	2.1	0.95
r21	31.37	31.15	0.7
r22	7.57	7.51	0.80

$$C = \frac{{Flux\left( {CaC{O_3}adjustment} \right) - Flux(N{H_3} \cdot {H_2}Oadjustment)}}{{Flux(N{H_3} \cdot {H_2}Oadjustment)}}$$

**Fig. 3:**
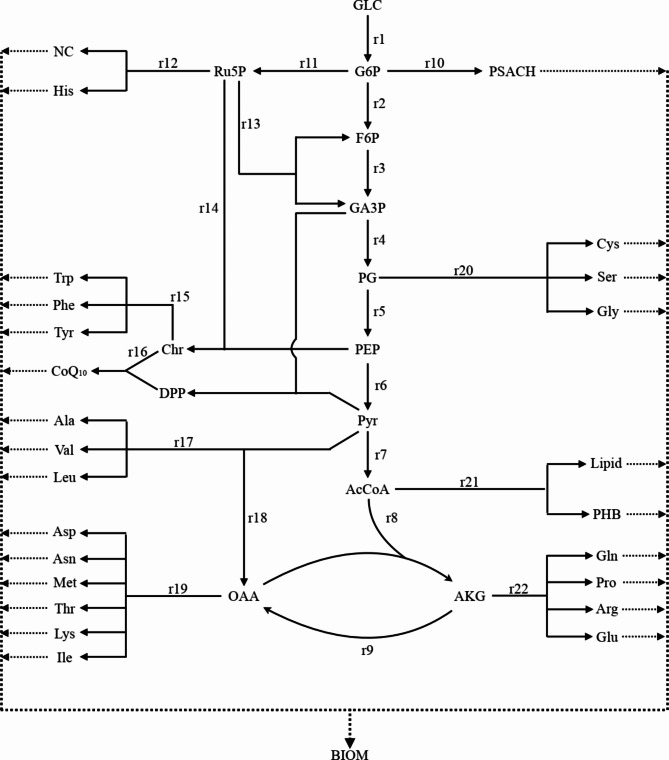
Metabolic network of CoQ_10_ synthesis in *R. sphaeroides.* AcCoA (Acetyl coenzyme A); AKG (a-Ketoglutarate); BIOM (biomass); Chr (Chorismate); F6P (Fructose-6-phosphate); G6P (Glucose-6-phosphate); GA3P (Glyceraldehyde-3-phosphate); GLC (Glucose); OAA (Oxaloacetate); PEP (Phosphoenolpyruvate); PG (3-Phosphoglycerate); PHB (poly-β-hydroxybutyrate); PSACH (Polysaccharides); PYR (Pyruvate); Ru5P (Ribulose-5-phosphate); NC (Nucleic Acids)

It can be seen from Fig. 3 and Table 2 that the two pH regulators caused significant changes in the flux distribution of G6P, Ru5P, and PEP nodes. The values of r2 to r9 on the EMP pathway and TCA pathway were smaller, while the values of r11 and r13 on the HMP pathway were larger when CaCO_3_ was used to adjust pH compared to NH_3_·H_2_O. This indicates that CaCO_3_ adjustment strengthened the HMP pathway and weakened the EMP pathway and TCA pathway.

The change in r16 showed that the metabolic flux from Chr to CoQ_10_ under CaCO_3_ adjustment was 0.13, while NH_3_·H_2_O adjustment resulted in 0.03. The enhanced metabolic flux from Chr to CoQ_10_ increased the demand for NADPH, prompting more G6P to flow into the HMP pathway to form NADPH. However, CaCO_3_ adjustment has little effect on biosynthesis. Thus, the increased portion of G6P entering the HMP pathway was not used for the synthesis of bacterial cells, but formed G6P and GA3P and returned to the EMP pathway for oxidative catabolism in the TCA pathway.

The organic acids produced during fermentation react with CaCO_3_ to form Ca^2+^, which activates enzymes related to the CoQ_10_ synthesis pathway and stimulates the production of reactive oxygen species (ROS) that can damage cell membranes [27]. Since CoQ_10_ has antioxidant properties, it can alleviate the damage caused by ROS on cell membranes. With the increase in Ca^2+^ concentration, the formation of intracellular ROS was stimulated, which promoted the synthesis of CoQ_10_ and increased the yield of CoQ_10_.

A certain amount of Ca^2+^ has been proven to increase the yield of CoQ_10_. Whether different concentrations of Ca^2+^ can all achieve this effect is an aspect worthy of future research, and perhaps the best Ca^2+^ concentration can be found. Ca^2+^ is an activator of many enzymes, and the hypothesis that increased CoQ_10_ yield may also be due to its activation of the activity of related enzymes in the synthesis pathway can also be explored.

### Changes in metabolic flux in G6P and Ru5P nodes

**Fig. 4:**
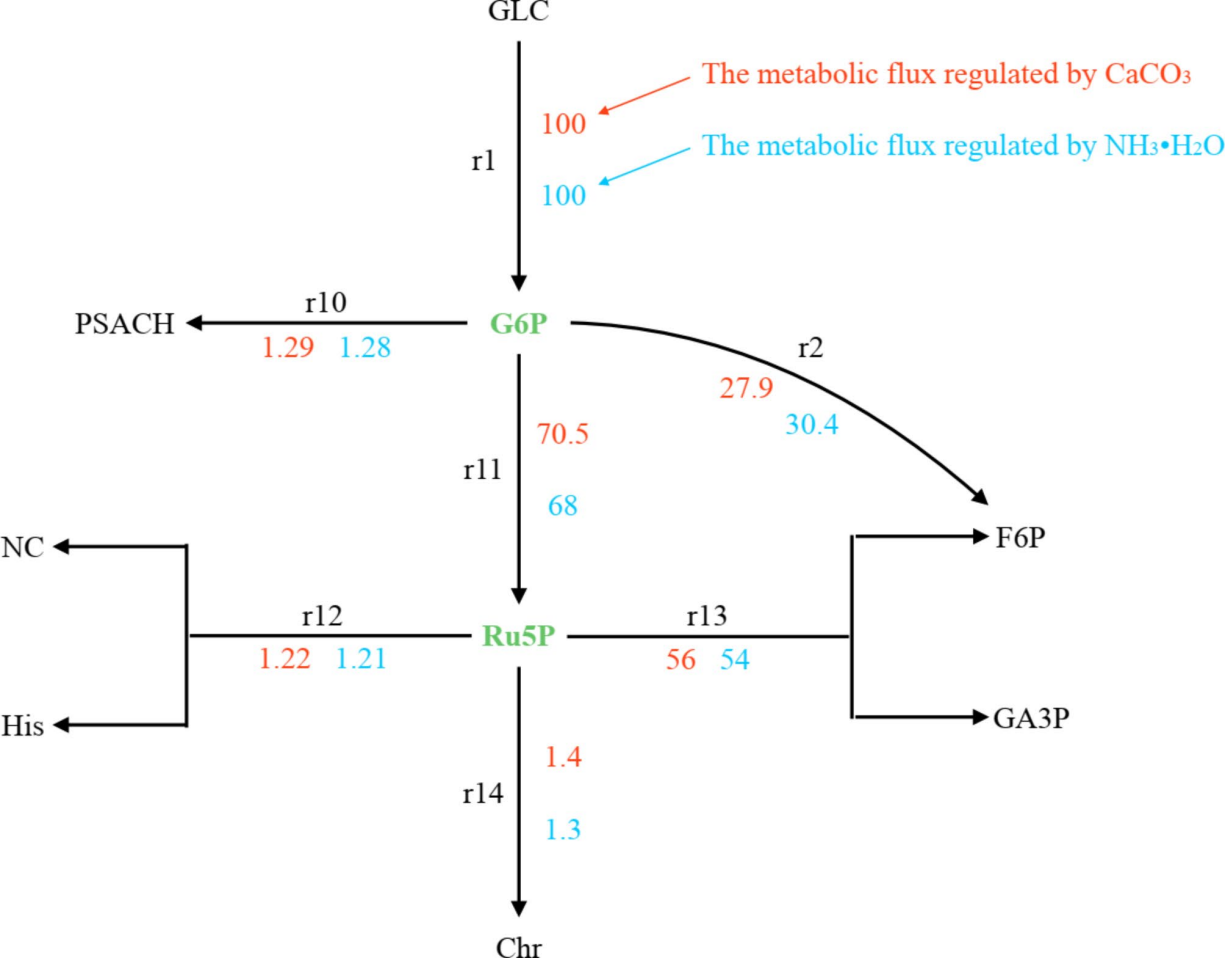
The metabolic flux distribution of G6P and Ru5P nodes under different pH adjustment modes

G6P is the common starting metabolite of both the EMP pathway and the HMP pathway. When CaCO_3_ was used to adjust the pH, the metabolic flux from G6P to Ru5P was 70.5, and the metabolic flux from G6P to F6P was 27.9 (Fig. 4). When NH_3_·H_2_O was used to adjust the pH, the metabolic flux from G6P to Ru5P was 68, and the metabolic flux from G6P to F6P was 30.4. The difference in metabolic flux caused by these two pH adjustment methods was equal between G6P to Ru5P and G6P to F6P. However, the increased metabolic flux of Ru5P had almost no effect on nucleic acid and histidine synthesis in the bacterium, which was mostly converted to F6P and GA3P (approximately 2.01). The metabolic flux from G6P to F6P migrates due to the addition of CaCO_3_ during fermentation or the effect of the reduction of NH_4_^+^ on fermentation. Some of the metabolic streams passed through the HMP pathway before entering the EMP pathway. This process has a completely different meaning than that of F6P, which is directly converted to F6P by the EMP pathway. Although the loss of carbon source material flux is small, the former provides more reducing power NADPH. Since the synthesis of isoprene pyrophosphate, the precursor of the CoQ_10_ side chain, requires NADPH to provide reducing power, 10 mol NADPH is directly involved for every 1 mol CoQ_10_ synthesis. Then, G6P enters the EMP pathway through the HMP pathway to provide NADPH for the synthesis of CoQ_10_.

### Analysis of the effects of two pH regulators on the synthesis of CoQ_10_ by *R. sphaeroides*

**Fig. 5:**
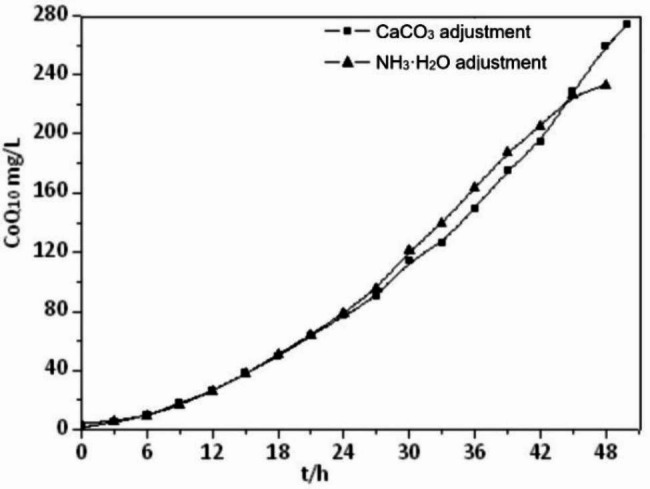
Effects of two pH regulators on the yield of CoQ_10_

The two pH regulators, CaCO_3_ and NH_3_·H_2_O, had almost no effect on the biosynthesis of the bacterium during the synthesis of CoQ_10_ by *R. sphaeroides*. The fermentation times used for conditioning with CaCO_3_ and NH_3_·H_2_O were 50 h and 45 h, respectively (Table 3). The difference in CoQ_10_ yield caused by the two pH regulators was significant (Fig. 5). When the fermentation endpoint was reached, the CoQ_10_ yield with CaCO_3_ conditioning was 274.43 mg·L^-1^ (8.71 mg·g^-1^ DCW), which was higher than that of NH_3_·H_2_O conditioning of 226.53 mg·L^-1^ (7.55 mg·g^-1^ DCW). The CoQ_10_ synthesis rates under the two pH adjustments were basically the same within 24 h from the beginning of fermentation. However, between 24 and 42 h, the rate of CoQ_10_ synthesis under NH_3_·H_2_O regulation was faster than that under CaCO_3_ regulation. The accelerated synthesis of CoQ_10_ in this process produces more organic acids. As an alkaline substance, NH_3_·H_2_O with high solubility can quickly resist the pH drop caused by organic acids, while CaCO_3_ with poor solubility reacts slowly. Therefore, NH_3_·H_2_O can better regulate the pH in this stage, which leads to a faster rate of CoQ_10_ synthesis. After 42 h of fermentation, the rate of NH_3_·H_2_O-regulated CoQ_10_ synthesis slowed down, whereas the CaCO_3_-regulated one was still able to maintain a high rate of growth. The reason was mainly that NH_3_·H_2_O was consumed in large quantities in the early stage, the concentration decreased dramatically, and the pH-regulating ability was weakened.

**Table 3:** Comparison of various parameters of the fermentation process under two pH adjustment methods

Parameters	CaCO_3_ adjustment	NH_3_·H_2_O adjustment
Initial glucose concentration (g·L^− 1^)	31.5	30
Fermentation time (h)	50	45
The yield of CoQ_10_ (mg·L^− 1^) (mg·g^− 1^ DCW)	274.43 8.71	226.53 7.55
The specific generation rate of CoQ_10_ (mg·g^− 1^·h^− 1^)	0.3681	0.3323
Glucose consumption rate (g·L^− 1^·h^− 1^)	0.63	0.67
Glucose ratio consumption rate (h^− 1^)	0.0439	0.0466
Production intensity (mg·L^− 1^·h^− 1^)	5.49	5.03
Average specific growth rate (h^− 1^)	0.0242	0.0250
Maximum cell dry weight (g·L^− 1^)	19.57	20.72

## Conclusions and future perspectives

In summary, pH adjustment with CaCO_3_ was more favorable for the synthesis of CoQ_10_ by *R. sphaeroides* compared with NH_3_·H_2_O adjustment. This study provides a reference for researchers in the selection of pH regulators. In addition, the main nodes of the metabolic network were identified as G6P and chorismic acid. This provides a theoretical basis for the modification of genes related to the CoQ_10_ synthesis pathway.

Due to the high value of CoQ_10_, an increasing number of researchers are investigating it. Its potential functions are constantly being developed and explored. Microbial fermentation as a production method will be the choice of more people in the future, and one of its most important goals is to maximize the yield of the desired fermentation product. The method is not only the well-known improvement and selection of strains, but also the analysis of the culture environment favorable to the synthesis of products according to the metabolic network of the microorganism, as used in this article. The application of metabolic engineering principles to improve the yield of microbial fermentation products has become a hot research topic.
